# Efficacy of Lower Limb Orthoses in the Rehabilitation of Children Affected by Cerebral Palsy: A Systematic Review

**DOI:** 10.3390/children11020212

**Published:** 2024-02-06

**Authors:** Sandra Miccinilli, Fabio Santacaterina, Rebecca Della Rocca, Silvia Sterzi, Federica Bressi, Marco Bravi

**Affiliations:** 1 Department of Physical and Rehabilitation Medicine, Fondazione Policlinico Universitario Campus Bio-Medico, Via Alvaro del Portillo, 200, 00128 Rome, Italy; s.miccinilli@policlinicocampus.it (S.M.); f.santacaterina@policlinicocampus.it (F.S.); rebecca.dellarocca@alcampus.it (R.D.R.); s.sterzi@policlinicocampus.it (S.S.); m.bravi@policlinicocampus.it (M.B.); 2 Research Unit of Physical and Reahabilitation Medicine, Department of Medicine and Surgery, Università Campus Bio-Medico di Rome, Via Alvaro del Portillo, 21, 00128 Rome, Italy; 3 Department of Engineering, Research Unit of Advanced Robotics and Human-Centred Technologies, Università Campus Bio-Medico di Rome, Via Alvaro del Portillo, 21, 00128 Rome, Italy

**Keywords:** cerebral palsy, AFO, KAFO, gait, hemiplegia, diplegia, balance

## Abstract

Lower limb orthoses are frequently used in children suffering from cerebral palsy (CP) alongside rehabilitation. The aim of this study was to analyze the effectiveness of ankle–foot orthosis (AFO) and knee–ankle–foot orthosis (KAFO) in walking, balance maintenance, spasticity, and quality of life improvement during rehabilitation in children affected by CP. The hypothesis was that the use of orthoses could improve the parameters compared to non-use. A systematic review was conducted in the main databases, including English language RCTs published about the use of AFO and KAFO in combination or not with rehabilitation methods in children affected by CP and studies mentioning walking, balance, muscle length, and quality of life as outcomes. From an initial number of 1484 results, a final number of 11 RCTs were included, comprising a total number of 442 participants and showing an overall high risk of bias in 10 studies and some concerns in one study. Six studies investigated the domain of walking, four studies investigated the domain of balance, and two studies investigated how KAFO and AFO orthoses could improve and prevent muscle contractures. Using highly heterogeneous study designs, different kinds of orthoses and different assessment tools were used. Further studies conducted with higher methodological quality are needed to establish whether AFO and KAFO are useful or not in combination with rehabilitation in improving the investigated domains.

## 1. Introduction

Cerebral palsy (CP) is a mobility and posture disorder resulting from non-progressive damage to the nervous system in the fetus or newborn. 

Mobility disorders, poor balance, and sensory deficits can be associated with cognitive problems, epilepsy, hip displacement, incontinence, dysphagia, behavior, and sleep disorders [1,2,3]. CP is commonly classified into spastic, dyskinetic, ataxic, or mixed according to the prevalent mobility disorder, being the spastic form the most common kind of presentation [2]. Spastic CP can further be classified into diplegic, hemiplegic, and quadriplegic, according to the number of affected limbs [3].

A child with CP frequently presents musculoskeletal disorders, resulting from poor motor control, abnormal biomechanical alignment, poor muscle activation, impaired agonist–antagonist balance, and balance disorders. For this reason, orthotics and aids are prescribed alongside the rehabilitation activity for the prevention and correction of any deformities, the maintenance of correct muscle lengths, and the improvement of postural control parameters [4].

Orthoses are used to support any impaired segment of the body, inhibit or increase its movement and improve function, prevent the development of contractures and deformities, maintain and stabilize extremities undergoing treatment in a functional position, support weak muscles, to improve selective motor control, reduce spasticity, and protect the extremities in the post-operative period. 

The sum of these advantages leads to a sensitive improvement in walking performance and energy expenditure. Several studies are reported in the literature showing that children affected by CP can benefit from AFO use since there are demonstrated benefits in spatial–temporal, kinematic, and kinetic gait parameters and energy expenditure reduction, as reported by Aboutorabi in 2017 [5]. More in depth, solid AFO (SAFO) neutralizes ankle angle and improves knee flexion angle, returning a faster walking speed with reduced energy expenditure [6]. The same effects are reported with hinged AFO (HAFO) use, which also prevents equine deformity and increases gait symmetry [7,8]. dynamic AFO (DAFO) seems to modify late stance and improve push off power at pre-swing, overall producing a better gait performance and also reducing energy expenditure [9]. This kind of AFO seems to be more suitable for children with a crouched gait pattern and equinus or dropped feet [9]. 

The key to building an effective orthosis is the comprehension of the musculoskeletal system and its internal and external forces [10].

Orthoses can also be used to improve standing and work on the perception of body weight distribution, with potential psychological and psychosocial benefits [5,11,12].

When treatment with orthotics is prescribed, particular attention is given to when and for how long they should be worn during the day. Adherence to this type of treatment is in fact greater among the child and family who clearly received the information [4].

Lower limb orthoses are usually classified into knee–ankle–foot orthoses (KAFOs) and ankle–foot–orthoses (AFOs). KAFOs are conceived to protect the articular range of motion, support impaired muscle, improve knee extension during standing and walking, and avoid knee hyperextension. The use of KAFO braces allows one to reach and maintain an upright position since knee and ankle joints are stabilized and allow the pelvis to develop strategies for posture control thanks to the correct alignment of the pelvis and lower limbs [13]. AFOs, instead, wrap the entire ankle and the whole foot or part of it [14]. AFOs are projected, instead, to improve the quality and energy expenditure in walking, enhance the functionality of the foot and ankle [4], promote the correct alignment of the ankle, and prevent muscle contractures by slowly stretching hypertonic muscles [15].

The aim of this systematic review of the literature was to analyze the effectiveness of the use of AFO and KAFO in the rehabilitation of children suffering from CP and how these can improve the parameters of balance and walking, contribute to the prevention of muscle contractures, and improve quality of life. The hypothesis was that the use of orthoses could improve such parameters compared to non-use.

## 2. Materials and Methods

### 2.1. Search Strategy

This systematic review was performed following the methodological indications of the PRISMA 2020 guidelines [16] in the following databases: the US National Library of Medicine (Pubmed), SCOPUS, Web of Science, (WOS), and Cochrane Database of Systematic Reviews. The study question was formulated according to the Population, Intervention, Comparison, and Outcome (PICO) model [17] (Table 1). For the search string, MeSH and free terms were used, in accordance with the specifications of the various databases consulted (Appendix A). This study was registered on the PROSPERO system with the number CRD42023445058.

### 2.2. Eligibility Criteria and Data Extraction

Studies meeting the following criteria were included in the review: English and Italian language studies; randomized clinical trials; studies including a population of children (<18 years) affected by CP treated with ankle–foot orthoses (AFOs) and/or knee–ankle–foot orthoses (KAFOs); and studies measuring results in the domains of walking, balance, prevention of muscle contractures, and quality of life.

Articles in languages other than English and Italian, articles that were not RCTs, articles including the adult population (>18 years), and articles considering orthoses other than AFOs and KAFOs were excluded. Duplicates were primarily detected and excluded via the Rayyan web app for systematic reviews [18].

The search was conducted without temporal limitations.

Two reviewers independently assessed titles and abstracts for eligibility. Subsequently, they discussed the inclusion of the articles they independently selected. At the end of this phase, the two reviewers evaluated the full text of the articles to verify their eligibility criteria. After choosing the studies, data were extracted. Any discrepancies or disagreements between the two reviewers that were highlighted in the previous phases were discussed and resolved with the help of a third reviewer.

### 2.3. Quality Assessment, Risk of Bias, and Evidence Synthesis 

The methodological quality of the included studies was assessed by two independent reviewers, and a third reviewer was consulted in case of disagreements to find a resolution. The Cochrane risk of bias tool (Rob2) [19] for randomized trials was used to assess the methodological quality of the included studies.

## 3. Results

### 3.1. Study Selection

**Figure 1:**
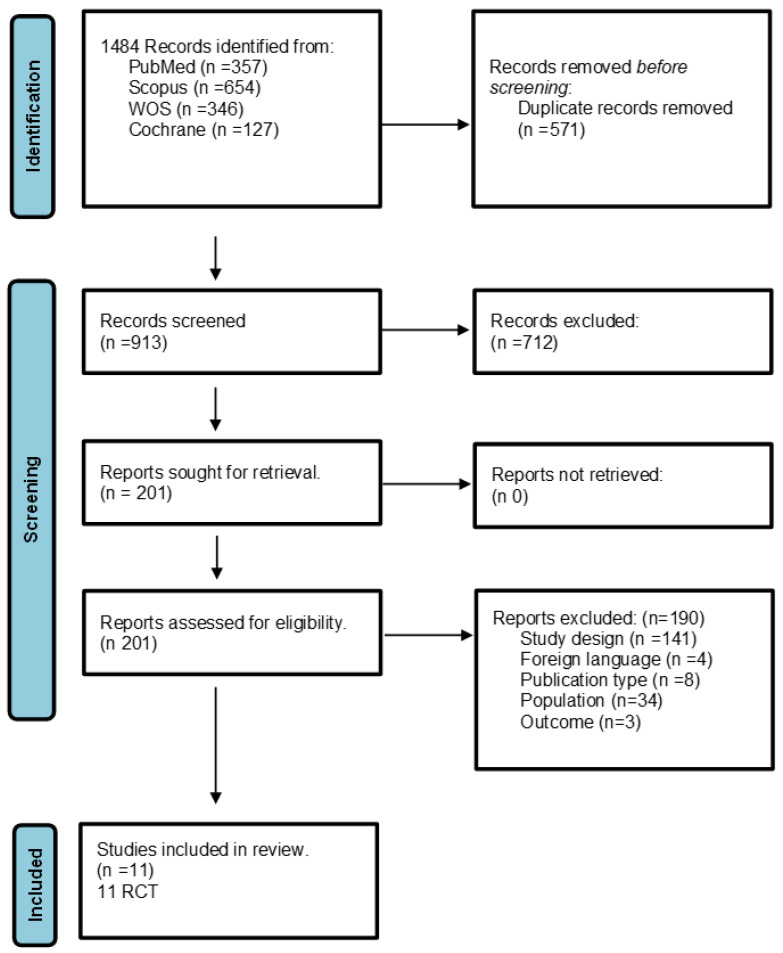
PRISMA flowchart: identification of studies via databases and registers.

A total number of 1484 studies were found after the systematic search of online databases. A total of 571 duplicates were excluded after the analysis by means of Rayyan web app (https://www.rayyan.ai/). The first screening phase through the analysis of title, abstract, and keywords led to the exclusion of 913 articles that did not meet the inclusion criteria. A total of 201 articles were, therefore, analyzed in detail to verify their eligibility. Eight studies were excluded due to the publication type (i.e., study protocol), four studies were excluded because they were written in a language other than English or Italian, 141 were excluded because the study design did not meet the inclusion criteria, 34 articles were excluded because they treated an adult population affected by CP (>18 years), and three articles were excluded because the outcomes did not meet the PICO question and the inclusion criteria. The research process is summarized in Figure 1 (PRISMA flowchart).

### 3.2. Study Characteristics

A total of 11 RCTs published between 2006 and 2022 were included. The main characteristics (author and year of publication, study design and purpose, participants, interventions, outcomes, and follow up) were summarized in Table 2. Studies were conducted in different countries around the world, including Italy (1), USA (3), Egypt (7), Holland (1), and China (1). A total number of 442 subjects affected by CP were enrolled in experimental or control groups. Six studies investigated how different types of braces, flexible, articulated, and rigid (hinged AFO, HAFO, carbon spring AFO, CAFO, dynamic AFO, DAFO, adjustable dynamic response AFO, ADR AFO, AFO, ground reaction AFO, GRAFO) could improve walking [20,21,22,23,24,25]. Four studies also investigated how DAFO, solid AFO, SAFO, AFO, and GRAFO could influence balance [23,26,27,28]. Two studies instead analyzed how KAFO and AFO could improve and prevent muscle contractures [28,29].

### 3.3. Quality of Included Studies

**Figure 2:**
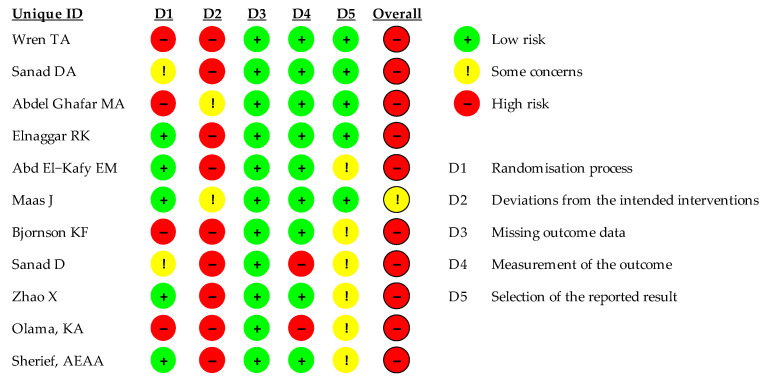
ROB2 results: risk of bias of the included RCTs.

The methodological quality of the included RCTs was assessed using the Cochrane Risk of Bias tool Rob2 (Review Manager RevMan) [19] [Computer program] Version 5.3. The following evaluated items are summarized in Figure 2: randomization process, deviations from the intended interventions, missing outcome data, measurement of the outcome, and selection of the reported result.

Studies were rated as having a high, low, or unclear risk of bias for each component of the tool. Finally, an overall result of a high risk of bias was assigned in twelve studies, and some unclear elements in one study (Figure 2).

## 4. Discussion

Lower limb orthoses are frequently used in children suffering from cerebral palsy (CP) alongside rehabilitation. The aim of this study was to analyze the effectiveness of AFO and KAFO in walking, balance maintenance, spasticity, and quality of life improvement during rehabilitation in children affected by CP.

Few reviews investigated the effectiveness of lower limb orthoses in CP and, to our knowledge, there is no systematic review in the literature investigating the use of both AFO and KAFO in children affected by CP. Aboudorabi in 2017, Firouzeh in 2021, and Autti Ramo in 2006, in fact, investigated the efficacy of AFO in improving gait parameters in CP-affected children and all concluded that higher quality studies are needed to draw conclusions and recommendations about the use of AFO [5,11,12]. No review investigated the state of the art about the use of KAFO for CP.

Studies included in this review showed a general improvement in motor functions, balance control, and gait spatial–temporal and kinematic parameters during AFO use compared to non-use. Despite this, the very high heterogeneity of the included studies did not allow the authors to identify a more effective AFO compared to the others, according to what was already assessed in the literature. For the same reason, the authors were unable to conduct a quantitative assessment and meta-analysis. 

More in detail, in the work of Wren et al [9], where DAFO and ADR-AFO are compared, it appears that they both improve hip extension in the stance phase and dorsal flexion of the ankle during the swing phase compared to walking barefoot. DAFOs improve ankle kinematics more during the final stance phase, whereas ADR-AFOs normalize energy expenditure more during the push off phase, consequently improving energy expenditure during walking. In the same study, children using DAFOs more than ADR-AFOs record a greater number of steps per day. In the questionnaires administered to parents, a preference for DAFOs due to greater comfort and lightness was found as well.

The effectiveness of DAFOs combined with treadmill training in improving the results of the Gross Motor Function Scale (GMFCS), in particular in static walking and balance, was also highlighted by Sherief [24]. Bjornson investigated in 2006 the effectiveness of DAFOs on the motor skills of children with CP, highlighting an improvement in the GMFCS score in the domains of crawling, kneeling, static balance, walking, running, and jumping [23].

Zhao [29] on the other hand, assessed no significant difference in GMFCS in night and day use of AFO braces, in terms of muscle strength and muscle lengths.

The literature, however, describes that a treatment performed with a particular type of AFO with triple lateral support can effectively improve balance and posture parameters [27]. For the balance parameter, another study by Sanad et al. in 2022 stated that GRAFOs were more effective in achieving and maintaining all stability indicators compared to classic AFOs. The authors explain this result, stating that AFOs help keep the ankle in a neutral position in static conditions; however, this causes movement restriction, and this effect is detrimental to balance control. GRAFOs, on the contrary, help create optimal alignment of the knee, stimulating its extension and thus improving postural control [25]. The same author, in 2018 [26], conducted a study comparing the immediate effect of SAFOs and GRAFOs on the balance of children with CP. Even in that case, GRAFOs were shown to be superior in maintaining and improving balance in the short term. In addition to this, GRAFOs used in combination with TheraTogs™ orthoses during the treatment of children with CP produced a more evident improvement in walking parameters compared to conventional rehabilitation treatment with or without TheraTogs™.

Other methods were compared to the use of orthoses to improve walking. In particular, Abdel Ghafar et al. [20] investigated the usefulness of the application of neuromuscular taping compared to the use of AFOs. According to the results of this study, neuromuscular taping could represent an excellent alternative to the use of AFOs for the short-term improvement of the spatiotemporal parameters of walking.

Elnaggar et al. [21] studied how shock waves and orthoses, combined with rehabilitation intervention, could have positive effects on walking, spasticity, and balance. The study demonstrated how a three-month treatment with shock waves and orthoses, accompanied by neuromotor rehabilitation interventions, did not lead to significant results in improving balance or walking in children with CP compared to rehabilitation alone. However, the combination of the two treatments was proven useful in reducing the spasticity of posterior chain muscles.

The only study considering the use of KAFO braces assessed that they are very poorly tolerated and uncomfortable and that they did not produce significant differences in the prevention of clubfoot in children with CP who wore them every night for a year, alternating legs, compared to children who not undergoing this kind of treatment [28]. 

In addition to the diversity of the proposed study designs, a high heterogeneity in the use of assessment tools in the domains of walking, spasticity, balance, and quality of life was found. Olama measured balance with the Biodex platform and tested it for dynamic balance [27], while Sanad only used the Biodex platform [19]. For the outcome measures used for spasticity, in Maas’ study [28], a digital inclinometer, a dynamometer, was used, and a video recording of patients walking and the GMFCS were performed. Differently, in Elnaggar’s study, electromyography was performed to measure the same parameter [21]. 

One of the main limitations of this systematic review was represented by the high variety of the analyzed orthoses and relative outcomes, which prevented authors from performing a metanalysis. In fact, within the RCTs included, seven different types of orthoses were adopted. Furthermore, a high heterogeneity of terminologies was used to describe AFOs, with consequent difficulties in study search and comparison. The outcome evaluation method was also found to be inconsistent. In addition to this, even if outcomes related to bodily functions were mostly investigated, no study investigated the psychological aspects and the level of participation of patients in the activities of daily and social life.

The included studies overall had a poor level of quality since the risk of bias assessment of the eleven RCTs included turned out a high risk of bias in ten studies and some unclear elements only in one study. In most studies, in fact, both the therapist and the patient were not blind to the kind of treatment carried out, and some studies did not carry out follow ups on the patients, describing only short-term results [20,23,26].

## 5. Limitations

The high heterogeneity of the included study designs, the variety of assessment tools, and the outcome measures prevented the authors from pooling data formation and from carrying out a metanalysis. Included RCTs mostly did not compare orthosis use versus non-use, whereas different kinds of orthoses were compared; therefore, no control group without orthoses was in most cases present, with a consequent low risk of bias. Further studies including participants with similar characteristics, where indications for orthotics use, previous use of botulin toxin, and surgeries are recorded, and more accurate research designs and methodologies are needed in order to assess whether the use of orthoses in CP-affected children is useful.

## 6. Conclusions

Nowadays orthoses are an integral part of the rehabilitation treatment of children with infantile CP, and they are, in fact, frequently prescribed to improve balance, walking, spasticity, and perceived quality of life. The results of this review show how in part of the included studies a general improvement in motor functions, walking, and balance emerge when braces are worn compared to non-use.

Despite this, further RCTs with less heterogeneous kinds of orthoses, shared outcome measures, and a lower risk of bias are still necessary to perform a metanalysis and draw conclusions about the actual effectiveness of orthoses use.

## Figures and Tables

**Table 1:** PICO question.

Patient, Population, or Problem	Intervention or Exposure	Comparison	Outcome
Children affected by CP	Orthosis use	Control group	Trunk control, balance, walking, prevention of muscle contractures, quality of life

**Table 2:** Synthesis of the included RCTs.

Author and Year	Design and Aim	Population	Interventions	Outcome and Follow Up	Results
Wren 2015 [9]	RCT Comparison of two different kinds of orthoses in children affected by CP	10 children affected by CP between 4 and 12 years of age with tiptoe walking or flexed knee deambulation	Participants wore DAFO and ADR-AFO for 4 weeks in a random order	StepWatch activity monitor, gait analysis, OPUS (Orthotics and Prosthetics Users’ Survey), PODCI (Pediatric Outcomes Data Collection Instruments) Barefoot evaluation at baseline, at 4 weeks, and at 8 weeks	Both orthoses improve step length, hip extension during the stance phase and foot dorsiflexion during the swing phase compared to barefoot evaluation. DAFO improves gait kinematics in the last stance phase and ADR-AFO improves gait kinematics during the initial swing phase. Parents preferred DAFOs since they were lighter and easier to use
Sanad 2022 [25]	RCT. AFO versus GRAFO for balance in diplegic CP children	30 children between 6 and 9 years of age	First group (n 15): AFO and rehabilitation for 3 months Second group (n 15): GRAFO and rehabilitation for 3 months	Biodex balance system at baseline and after 3 months	GRAFO is significantly better than AFO in balance control
Abdel Ghafar 2021 [20]	Single-blind RCT neuromuscular taping versus AFO in gait parameters improvement	39 children with spastic CP. Level l o ll GMF and hyperton 1 or 1+ on the Ashworth Scale	Control group (n 11) AFO treatment group (n 12) Taping group (n 13)	GAITRite System evaluation at baseline and after 4 weeks	Significant improvement in velocity, step length, and single support phase in both AFO and taping groups with respect to baseline
Elnaggar 2019 [21]	Single-blind RCT. Shock waves and orthoses therapy combined with physiotherapy in CP children with spastic diplegia	53 children between 5 and 8 years of age with CP, diplegia, spasticity between 1 and 1+ in MAS, and level I or II in GMF	First group (n 18): rehabilitation and shock waves Second group (n 16): rehabilitation and orthoses Third group (n 19): rehabilitation, orthoses, and shock waves	EMG, ePediatric Balance Scale, 3D-motion capture system Evaluations at baseline and after 3 months	No significant differences in gait and balance improvement Significant improvement in spasticity reduction in the third group
Ehab Mohamed Abd El-Kafy 2014 [22]	RCT. Orthotics correction impact on lower limb deformity in children affected by CP	57 children affected by CP that are 6 to 8 years of age	First group (n 18): rehabilitation with stance and gait exercises without orthoses Second group (n16): rehabilitation with stance and gait exercises with TheraTogs™ orthoses for both lower limbs Third group (n 17): rehabilitation with stance and gait exercises with TheraTogs™ orthoses and GRAFO Treatment length: 12 weeks	Gait analysis, ROM measurement in hip and knee rotation during stance phase, and gait parameters measurement (step length, velocity, cadence) Evalutaion before and after treatment	Combined use of GRAFO and TheraTogs™, during rehabilitation gives better results than rehabilitation without orthoses or with TheraTogs™
Maas 2014 [28]	Single-blind RCT. Efficacy and tolerance of KAFO in clubfoot prevention in CP	Children of 4 to 16 years of age affected by CP are able to walk	First group (n 15): usual rehabilitation + KAFO night use one leg at a time for one year Second group (n 13): control group, usual rehabilitation, no KAFO	Digital inclinometer (for ROM in dorsiflexion measurement) together with a dynamometer, video recording of gait and knee, foot and ankle for ROM measurement during demabulation, GMFM Evaluations at baseline and at 3, 6, 9, and 12 months	No significant differnces. Kafo is not well tolerated during sleep
Bjorson 2006 [23]	RCT. DAFO efficacy on motricity	23 children affected by CP between 12 months and 8 years of age having used DAFO for at least 4 h for one month	Two measurements one after the other with and without DAFO on the same day	GMFM-88-66 (crawling, kneeling down, standing, walking, running, jumping)	Significant increase in motricity
Sanad 2018 [26]	RCT. Immediate effect of SAFO vs. GRAFO on the balance of diplegic spastic children affected by CP	30 children affected by CP, diplegia, and spasticity between 5 and 8 years of age	First group (n 15): SAFO wearing Second group (n 15): GRAFO wearing	Biodex balance Board barefoot and with orthoses	GRAFO is better than SAFO in balance improvement
Zhao 2013 [29]	Single-blind RCT. Day use vs. day and night use of AFO in spastic diplegic CP children	112 spastic diplegic CP children	First group (n 53): daily AFO Second group (n 52): AFO night and day Both groups performed the usual rehabilitative treatment 5 days a week for 8 weeks	EMG, dynamometer, GMFM, passive angle measurement. Evaluation was performed at baseline and the end of treatment (8 weeks)	No significant differences between the two groups
Olama 2013 [27]	RCT. Three sides supported AFO’s role in balance improvement in children affected by CP and spastic diplegia	30 children affected by CP and spastic diplegia	First group (n 15): AFO and rehabilitation for 30 min, three times per week for 6 months Second group (n 15): rehabilitation	Ashworth Scale for spasticity assessment, dynamic balance test with a Biodex balance board for balance assessment Evaluations at baseline and after 6 months	Improvement of posture and balance with AFO
Sherief 2015 [24]	RCT. Efficacy of treadmill treatment combined with DAFO in balance improvement in CP emiplegic children	30 spastic emiplegic children affected by CP	First group (n 15): rehabilitation for 60 min, three times per week for three months Second group (n 15): rehabilitation for 60 min, three times per week for three months + training on a treadmill wearing DAFO for 30 min	Evaluation at baseline and after three months with The Peabody Developmental Test of Motor Proficiency and a Biodex balance board	12 weeks of treatment with treadmill, and DAFO improves gross motor function (standing and walking) and balance

Legend: hinged AFO: HAFO; carbon spring AFO: CAFO; dynamic AFO: DAFO; adjustable dynamic response AFO: ADR AFO; ground reaction AFO: GRAFO; solid AFO: SAFO.

## Data Availability

The data presented in this study are available on request from the corresponding author. The data are not publicly available due to no new data were created or analyzed in this study.

## Appendix A

### Search Strategy

Database: Pubmed; terms used for the search: “Cerebral Palsy” [MeSH Terms] OR “cerebral pals*” [All Fields] OR (“Cerebral Palsy” [MeSH Terms] OR (“cerebral” [All Fields] AND “palsy” [All Fields]) OR “Cerebral Palsy” [All Fields] OR (“monoplegic” [All Fields] AND “infantile” [All Fields] AND “cerebral” [All Fields] AND “palsy” [All Fields])) OR (“Cerebral Palsy” [MeSH Terms] OR (“cerebral” [All Fields] AND “palsy” [All Fields]) OR “Cerebral Palsy” [All Fields] OR (“cerebral” [All Fields] AND “palsy” [All Fields] AND “quadriplegic” [All Fields] AND “infantile” [All Fields])) OR (“Cerebral Palsy” [MeSH Terms] OR (“cerebral” [All Fields] AND “palsy” [All Fields]) OR “Cerebral Palsy” [All Fields] OR (“cerebral” [All Fields] AND “palsy” [All Fields] AND “rolandic” [All Fields] AND “type” [All Fields])) OR “Congenital Cerebral Palsy” [All Fields] OR “Little’s Disease” [All Fields] OR “Spastic Diplegia” [All Fields] OR “Athetoid Cerebral Palsy” [All Fields] OR “Dyskinetic Cerebral Palsy” [All Fields] OR “Atonic Cerebral Palsy” [All Fields] OR “cerebral palsy hypotonic” [All Fields] OR (“Cerebral Palsy” [MeSH Terms] OR (“cerebral” [All Fields] AND “palsy” [All Fields]) OR “Cerebral Palsy” [All Fields] OR (“cerebral” [All Fields] AND “palsy” [All Fields] AND “diplegic” [All Fields] AND “infantile” [All Fields])) OR “cerebral palsy spastic” [All Fields]) AND (“orthotic devices” [MeSH Terms] OR “device orthotic” [All Fields] OR “Orthosis” [All Fields] OR “Parapodium” [All Fields] OR “orthotic devic*” [All Fields]) AND (“Pain” [MeSH Terms] OR “pain burning” [All Fields] OR “Physical Suffering” [All Fields] OR “pain migratory” [All Fields] OR “radiating pain” [All Fields] OR “Splitting Pain” [All Fields] OR “Ache” [All Fields] OR “Crushing Pain” [All Fields] OR “Pain” [All Fields] OR “Step” [All Fields] OR “March” [All Fields] OR “Walk” [All Fields] OR “gait” [All Fields] OR “Postural Balance” [MeSH Terms] OR “Posture Equilibrium” [All Fields] OR “balance postural” [All Fields] OR “Posture Balance” [All Fields] OR “Musculoskeletal Equilibrium” [All Fields] OR “Postural Control” [All Fields] OR “control posture” [All Fields] OR “quality of life” [All Fields] OR “Health-Related Quality of Life” [All Fields])

Database: Scopus; terms used for the search: (“Cerebral Palsy” OR “Cerebral Pals*” OR “Monoplegic Infantile Cerebral Palsy” OR “Cerebral Palsy, Quadriplegic, Infantile” OR “Cerebral Palsy, Rolandic Type” OR “Congenital Cerebral Palsy’’ OR ‘’ Little’s Disease” OR “Spastic Diplegia” OR “Athetoid Cerebral Palsy” OR “ Dyskinetic Cerebral Palsy” OR “Atonic Cerebral Palsy” OR “Cerebral Palsy, Hypotonic” OR “Cerebral Palsy, Diplegic, Infantile” OR “Cerebral Palsy, Spastic” ) AND (“orthotic devices” OR “Device, Orthotic” OR “Orthosis” OR “Parapodium” OR “orthotic devic*”) AND (“Pain” OR “Pain, Burning” OR “Physical Suffering” OR “Pain, Migratory” OR “Radiating, Pain” OR “Splitting Pain” OR “Ache” OR “Crushing Pain” OR “Pain” OR “Step” OR “March” OR “Walk”OR “gait” OR “Postural Balance” OR “Posture Equilibrium” OR “Balance, Postural” OR “Posture Balance” OR “Musculoskeletal Equilibrium” OR “Postural Control” OR “Control, Posture” OR “quality of life” OR “Health-Related Quality of Life”)

Database: Web Of Science; terms used for the search: (“Cerebral Palsy” OR “Cerebral Pals*” OR “Monoplegic Infantile Cerebral Palsy” OR “Cerebral Palsy, Quadriplegic, Infantile” OR “Cerebral Palsy, Rolandic Type” OR “Congenital Cerebral Palsy’’ OR ‘’ Little’s Disease” OR “Spastic Diplegia” OR “Athetoid Cerebral Palsy” OR “ Dyskinetic Cerebral Palsy” OR “Atonic Cerebral Palsy” OR “Cerebral Palsy, Hypotonic” OR “Cerebral Palsy, Diplegic, Infantile” OR “Cerebral Palsy, Spastic” ) AND (“orthotic devices” OR “Device, Orthotic” OR “Orthosis” OR “Parapodium” OR “orthotic devic*”) AND (“Pain” OR “Pain, Burning” OR “Physical Suffering” OR “Pain, Migratory” OR “Radiating, Pain” OR “Splitting Pain” OR “Ache” OR “Crushing Pain” OR “Pain” OR “Step” OR “March” OR “Walk”OR “gait” OR “Postural Balance” OR “Posture Equilibrium” OR “Balance, Postural” OR “Posture Balance” OR “Musculoskeletal Equilibrium” OR “Postural Control” OR “Control, Posture” OR “quality of life” OR “Health-Related Quality of Life”).

Database: Cochrane; terms used for the search: (“Cerebral Palsy” (MeSH Terms) OR “Cerebral Pals*” OR “Monoplegic Infantile Cerebral Palsy” OR “Cerebral Palsy, Quadriplegic, Infantile” OR “Cerebral Palsy, Rolandic Type” OR “Congenital Cerebral Palsy” OR “Little’s Disease” OR “Spastic Diplegia” OR “Athetoid Cerebral Palsy” OR “ Dyskinetic Cerebral Palsy” OR “ Atonic Cerebral Palsy” OR “Cerebral Palsy, Hypotonic” OR “Cerebral Palsy, Diplegic, Infantile” OR “Cerebral Palsy, Spastic” ) AND (“orthotic devices” OR “Device, Orthotic” OR “Orthosis” OR “Parapodium” OR “orthotic devic*”) AND (“Pain” (MeSH Terms) OR “Pain, Burning” OR “Physical Suffering” OR “Pain, Migratory” OR “Radiating, Pain” OR “Splitting Pain” OR “Ache” OR “Crushing Pain” OR “Pain” OR “Step” OR “March” OR “Walk”OR “gait” OR “Postural Balance” (MeSH Terms) OR “Posture Equilibrium” OR “Balance, Postural” OR “Posture Balance” OR “Musculoskeletal Equilibrium” OR “Postural Control” OR “Control, Posture”OR “quality of life” OR “Health-Related Quality of Life”).
